# The readability of parent information leaflets in paediatric studies

**DOI:** 10.1038/s41390-023-02608-z

**Published:** 2023-04-29

**Authors:** Elizabeth Nash, Matthew Bickerstaff, Andrew J. Chetwynd, Daniel B. Hawcutt, Louise Oni

**Affiliations:** 1 Department of Women’s and Children’s Health, Institute of Translational Medicine, University of Liverpool, member of Liverpool Health Partners, Liverpool, UK; 2 NIHR Alder Hey Children’s Clinical Research Facility, Alder Hey Children’s NHS Foundation Trust, Liverpool, UK; 3 Department of Paediatric Pharmacology, Alder Hey Children’s NHS Foundation Trust, Liverpool, UK; 4 Department of Paediatric Nephrology, Alder Hey Children’s NHS Foundation Trust, Liverpool, UK

## Abstract

**Background:**

Poor literacy can impact achieving optimal health outcomes. The aim of this project was to assess the readability of parent information leaflets (PILs).

**Methods:**

A single-centre study using paediatric PILs. Five readability tests were applied (Gunning Fog Index (GFI), Simple Measure of Gobbledygook (SMOG), Flesch Kincaid Grade Level (FKGL), Coleman–Liau Index (CLI) and Automated Readability Index (ARI)). Results were compared to standards and by subtype.

**Results:**

A total of 109 PILs were obtained; mean (±SD) number of characters was 14,365 (±12,055), total words 3066 (±2541), number of sentences 153 (±112), lexical density 49 (±3), number of characters per word 4.7 (±0.1), number of syllables per word 1.6 (±0.1) and number of words per sentence 19.1 (±2.5). The Flesch reading ease score was 51.1 (±5.6), equating to reading age 16–17 years. The mean PIL readability scores were GFI (12.18), SMOG (11.94), FKGL (10.89), CLI (10.08) and ARI (10.1). There were 0 (0%) PILs classed as easy (score <6), 21 (19%) mid-range (6–10) and 88 (81%) were difficult (>10). They were significantly above the recommended reading age (*p* < 0.0001) and commercial studies were least accessible (*p* < 0.01).

**Conclusion:**

Existing PILs are above the national reading level. Researchers should use readability tools to ensure that they are accessible.

**Impact:**

- Poor literacy is a barrier to accessing research and achieving good health outcomes.
- Current parent information leaflets are pitched far higher than the national reading age.
- This study provides data to demonstrate the reading age of a large portfolio of research studies.
- This work raises awareness of literacy as a barrier to research participation and provides tips on how to improve the readability of patient information leaflets to guide investigators.

## Introduction

Health literacy is the ability of individuals to access, understand, and use information in order to promote and maintain good health.^1^ Insufficient health literacy is linked to difficulty with comprehension of health information, limited disease knowledge, and lower adherence to medication.^2^ These contribute to a range of issues including ineffective healthcare use, ongoing poor health, increased costs, higher risks of mortality, and health disparities in those less literate.^3^ Reading ability is a crucial marker of health literacy. Those with inadequate literacy have difficulty reading and understanding material written with a reading age of 11–12 years, whilst those with marginal literacy have difficulty understanding the material for age 15–16 years.^4^ A national survey found that in the United Kingdom, around 1 in 6 people have levels of general literacy below that expected of an 11-year old.^5^ The National Literacy Trust estimated that 16.4% of adults in England, equating to 7.1 million people, are functionally illiterate.^6^ This means that they have a reading age of 11 years or below and they can only comprehend straightforward, short texts on familiar topics.

In clinical settings, healthcare jargon adds complexity to reading with a previous study highlighting that 43% of written health information was too complicated for UK adults to fully comprehend.^1^ This figure increases to 61% when numerical information is added, for example, one in three adults were unable to understand basic usage instructions on a medicine label and adults with low literacy were twice as likely to die when compared to those with adequate literacy.^7^

Readability formulas can objectively evaluate written health information by calculating the number of formal years of schooling a reader requires in order to understand the material.^8^ Amongst the available tools are the Gunning Fog Index (GFI), Simple Measure of Gobbledygook (SMOG), Flesch Kincaid Grade Level (FKGL), Coleman–Liau Index (CLI) and the Automated Readability Index (ARI). Each formula uses different criteria to determine a reading age and it is recommended when using multiple readability formulas to assume the highest calculated reading age or an average across the tools.^8,9^ A previous study investigated the frequency of readability formulae used in the healthcare literature between 2005 and 2008 and found that the most used readability formulas were the FKGL (57.42%), the Flesch Reading Ease (44.52%) and the SMOG (25.81%).^8^

Paediatric research studies provide written health information prior to obtaining informed consent through the use of parent information leaflets (PILs)^9^. Parents are required to fully understand the information presented to them in order to make a decision on behalf of their child and appropriately written material is therefore of great importance.

The primary aim of this project was to analyse the readability of PILs using a portfolio of paediatric studies and to evaluate how this compares to the national health literacy levels. The secondary aim was to evaluate whether there was a difference in the readability of PIL between specialities and study subtype.

## Method

### Setting

The study was a single-centre cohort study undertaken at Alder Hey Children’s NHS Foundation Trust Hospital, Liverpool, UK.

### Eligible studies

An active clinical trial portfolio list was obtained on 4 July 2022. All studies that were currently open to recruitment were included, and all clinical trials or studies that were closed to recruitment (even if they remained open for follow-up data) were excluded.

### Regulatory approvals

Ethical approval was not needed for this study as it involved a secondary review of existing literature as per the National Health Service Research Authority guidance. The study was registered with the NIHR Alder Hey Clinical research facility senior management team and formed part of a social inequality work stream.

### Readability software and data collection

An online tool was used (Tests Document Readability, 2022 https://www.online-utility.org/english/readability_test_and_improve.jsp) to analyse the PILs for each study which reported the readability score obtained from the GFI, SMOG, FKGL, CLI and ARI tools. The advantages and disadvantages of each of the tools are outlined in Table 1. Some studies had multiple, often similar, PILs within the study due to >1 eligible patient group; in these circumstances, the investigators (E.N., L.O.) selected the PIL that was deemed most relevant for the study for analysis to avoid duplication. All text on the PIL was evaluated including contact information and data regulatory text. The consent forms were not analysed. For each PIL, the speciality was recorded, whether it was a medical or surgical study and the subtype of study in terms of commercial or non-commercial research. The number of characters, a number of total words, number of sentences, lexical density (the proportion of lexical words divided by the total number of words), average number of characters per word, average number of syllables per word and average number of words per sentence were recorded together with the Flesch reading ease score (the original reading age tool developed; designed based on the average sentence length and average number of syllables with a score 90–100 being easily understandable, score 60–70 equivalent to 8th/9th-grade education and score 0–30 equivalent to university grade education). The reading age was recorded to provide five readability test results (GFI, SMOG, FKGL, CLI, ARI).

**Table 1:** A summary of the advantages and disadvantages of each of the readability scoring tools.

Scoring tool	Advantages	Disadvantages
GFI	Considered to be an accurate readability formula	Omits that not all multi-syllabic words are difficult
SMOG	Used often in healthcare literature due to high consistency and ability to predict 100% comprehension	Cannot be used to calculate reading grade levels in languages other than English
FKGL	Accessible and easy to use. Readily available within Microsoft Office suite	Tends to predict lower reading grade levels
CLI	Relies on characters instead of syllables per word. Characters are more accurately counted mechanically compared to syllables	Designed for English language so may not be accurate in non-English text
ARI	Like CLI, ARI uses characters per word rather than syllables per word which is more readily counted by computer programs	May not be accurate for the use of evaluating the readability of non-English texts

*GFI* Gunning Fog Index, *SMOG* Simple Measure of Gobbledygook, *FKGL* Flesch Kincaid Grade Level, *CLI* Coleman–Liau Index, *ARI* Automated Readability Index.

### Data interpretation and statistical analysis

The readability tests compute a score that equates to the American grade level for education. Sixth grade corresponds to the sixth year of UK schooling, which can be translated to the age of 11–12, as shown in Table 2. As such, scores of 6–6.9 should be readable by the average 11–12-year old. Tenth grade corresponds to the tenth year of schooling, which can be deduced to be the age of 15–16 years and a readability score of 10.

**Table 2:** The American schooling grades used to produce a readability age by the tools, translated to the corresponding student age.

Age (years)	American school grade equivalent	Readability score
7–8	Grade 2	2
8–9	Grade 3	3
9–10	Grade 4	4
10–11	Grade 5	5
11–12	Grade 6	6
12–13	Grade 7	7
13–14	Grade 8	8
14–15	Grade 9	9
15–16	Grade 10	10
16–17	Grade 11	11
17–18	Grade 12	12

Readability scores for the purposes of this study were categorised into difficulty levels as follows:

- Easy: a score <6, equivalent to <6th grade or 6th year of schooling, aged 11–12 years.
- Average: a score of 6–10, equivalent to between 6th and 10th grade or 6th and 10th year of schooling, aged 12–16 years.
- Difficult: a score >10, more than 10th grade or 10+ years of schooling, aged 16+ years.

Data were tested for normality via Shapiro–Wilk test and normally distributed data were assessed for significance via unpaired *t*-tests, whereas non-normally distributed data were assessed via Mann–Whitney *U* tests using GraphPad Prism version 8.1.1. Cronbach’s alpha test was conducted in R studio in order to test for internal consistency. For each PIL, the readability scores were determined using the five different readability tools in their ability to establish the recommended reading level. A threshold score of 6.9 was taken as an acceptable level to align with previous literature suggesting health information should be pitched at a reading level less than the age of 11 years.^10^ Each tool’s results were then compared to this acceptable level. A *p* value of <0.05 was used to determine any statistical significance.

## Results

### Description of eligible studies

There were 174 studies identified on the active clinical trial portfolio at Alder Hey Children’s NHS Foundation Trust Hospital that were assessed for eligibility. Clinical trials or studies that were no longer open for recruitment were excluded (44 studies). The number of studies eligible were 134 (134/178; 75%). Using this list, PILs were obtained by the clinical research operational team or by contacting the relevant research nurse or principal investigators. Out of the 134 open studies, 109 PILs were obtained (81%). Of the included paediatric studies, the specialities were grouped as 18 haematology/oncology, 16 rheumatology, 9 orthopaedic/spinal surgery, 7 respiratory, 7 paediatric medicine specific, 7 psychology/mental health, 6 infectious diseases and microbiology, 5 endocrine, 5 renal, 5 critical care, 3 neurology, 3 neurosurgery, 3 paediatric surgery and urology, 2 ophthalmology, 2 gastroenterology, 2 general paediatric, 2 emergency medicine, 2 therapies (physiotherapy and speech and language), 1 developmental paediatrics, 1 cardiology, 1 diabetes, 1 cleft and dental/oral health, and 1 study was palliative care. The PILs were grouped into 93 (85%) medical studies and 16 (15%) surgical studies. There were 18 (17%) commercial studies and 91 (83%) were non-commercial studies.

### Readability scores according to each tool

Overall, the eligible cohort of PILs had a mean ± SD number of characters of 14,365 (±12,055), a number of total words of 3066 (±2541), a number of sentences of 153 (±112), a lexical density of 49 (±3) the average number of characters per word of 4.7 (±0.1), the average number of syllables per word of 1.6 (±0.1) and an average number of words per sentence of 19.1 (±2.5). The Flesch reading ease score was 51.1 (±5.6), equating to around a grade 11 reading ability (equivalent to grade 11; 16–17-year old).

The overall mean readability scores of the PILs are shown in Table 3. The average score across the tools was 11.0 (equivalent to grade 11; 16–17-year old). As can be seen, the mean scores ranged from a minimum score of 10.1 (equivalent to grade 10; 15–16-year old) to a maximum score of 12.2 (equivalent to grade 12; 17–18-year old) across the five tools. Cronbach’s alpha analysis between the five readability tools found there to be significant internal consistency between the readability tools (*p* = 0.97, 95% confidence interval of 0.096, 0.976).

**Table 3:** The overall mean, standard deviation (SD) and standard error (SE) of the PIL readability scores using the Gunning Fog Index (GFI), SMOG, Flesch Kincaid Grade Level (FKGL), Coleman–Liau Index (CLI) and Automated Readability Index (ARI).

	GFI	SMOG	FKGL	CLI	ARI
Mean	12.2	11.9	10.9	10.1	10.1
SD	1.3	0.9	1.3	0.9	1.6
SE	0.1	0.09	0.1	0.09	0.2
School grade	12	11	10	10	10
Age (years)	17–18	16–17	15–16	15–16	15–16

**Fig. 1:**
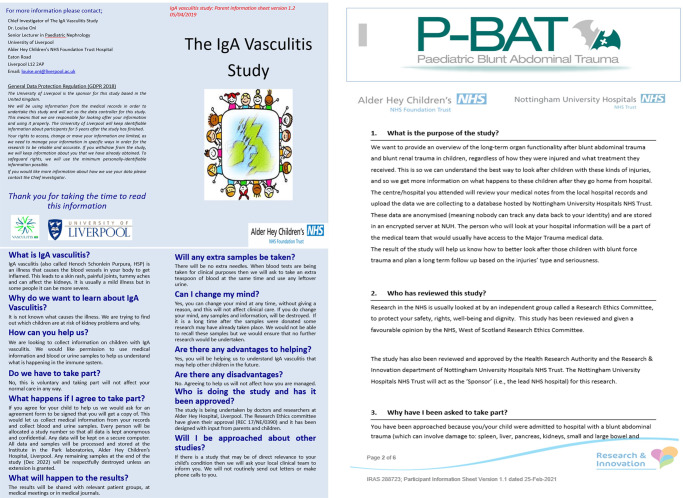
An example of two patient information leaflets (PIL) from the IgA vasculitis study and the paediatric blunt abdominal trauma study. These studies achieved an average score in terms of readability assessment and were deemed some of the most readable PIL in the cohort. For example, the overall average readability score of the paediatric blunt abdominal trauma PIL scored 8.6 (equivalent to grade 8 of schooling; 13–14 years old).

The PILs were divided into difficulty levels; there were 0 (0%) PILs in the easy (<6) range, 21 (19%) PILs in the average (6–10) range, and 88 (81%) in the difficult (>10) range. Apart from one study that achieved a score of 6.7 (equivalent to grade 6; 11–12-year old) using the ARI score, all of the PILs achieved readability scores above the predefined acceptable range of >6.9. The PIL with the lowest, and thus most accessible, overall readability score was a non-commercial, multi-centre national paediatric surgical study evaluating the long-term organ functionality after blunt abdominal trauma and blunt renal trauma in children (Fig. 1). It achieved an overall average readability score of 8.6 (equivalent to grade 8; 13–14-year old) across the five tools and it was the only study to achieve the acceptable reading score when evaluated using the ARI tool. In comparison, the PIL with the highest, thus most inaccessible, readability score was a commercial gastroenterology study looking into the treatment of eosinophilic oesophagitis with budesonide. It achieved an overall average readability score of 14.2 (equivalent to grade 14; >18 years old, degree level of education). It also achieved the highest overall individual readability score using the GFI tool with a score of 15.7 2 (equivalent to grade 15; >18 years old, degree level of education).

### Comparison of PILs analysis against recommended reading level

The mean value of each readability formula was compared. A Shapiro–Wilk test found the data to be normally distributed (GFI (*p* = 0.55), SMOG (*p* = 0.42), FKGL (*p* = 0.29), CLI (*p* = 0.07), ARI (*p* = 0.60)). A one-way *t*-test was performed to compare each readability test against the recommended reading level (mu = 6.9). The mean scores of each of the readability tools were all statistically significantly different from the recommended reading level (all *p* < 0.0001).

### Comparison of the readability of PIL according to medical or surgical speciality

A comparison was made between the PIL readability scores according to whether they were a medical (*n* = 93) or a surgical (*n* = 16) speciality study. There was no statistically significant difference in the readability scores between medical or surgical studies using any of the five tools (*p* > 0.05). There was a statistically significant difference in terms of the number of characters with medical studies having a greater number of characters (medicine mean 15,317 (±12,715), surgical 8832 (±4057), *p* = 0.04) and the average number of overall words used (medicine 3269 (±2679), surgical 1888 (±865), *p* = 0.03). There was no statistically significant difference between the Flesch reading ease score, the average number of sentences, lexical density, number of characters per word, number of syllables per word or number of words per sentence between the PILs when divided according to medical or surgical studies (all *p* > 0.05).

### Comparison of the readability of PIL according to commercial and non-commercial subtype

In order to determine if there was any difference between the study subtypes, the commercial (*N* = 18) and non-commercial studies (*N* = 91) were compared. There were statistically significant differences in all of the readability scores when comparing commercial studies with non-commercial studies, as shown in Table 4. The Flesch reading ease score and parameters within the text were also statistically different between the commercial and non-commercial studies (Table 4).

**Table 4:** The mean (SD) readability scores between commercial studies PILs when compared to non-commercial studies PILs.

Parameter	Commercial PIL (mean ± SD)	Non-commercial PIL (mean ± SD)	Statistical difference
GFI	13.4 ± 1.1	11.9 ± 1.2	*p* < 0.01
SMOG	12.9 ± 0.8	11.8 ± 0.8	*p* < 0.01
FKGL	12.3 ± 1.2	10.6 ± 1.1	*p* < 0.01
CLI	10.6 ± 0.9	10.0 ± 1.1	*p* < 0.01
ARI	11.8 ± 1.4	9.8 ± 1.4	*p* < 0.01
Number of characters	33,212 ± 17,667	10,637 ± 5508	*p* < 0.0001
Number of words	7027 ± 3728	2283 ± 1169	*p* < 0.0001
Number of sentences	318 ± 170	121 ± 55	*p* < 0.0001
Lexical density	53.0 ± 2.1	48.7 ± 2.5	*p* < 0.0001
Number of characters per word	4.7 ± 0.14	4.7 ± 0.13	*p* = 0.05
Number of syllables per word	1.64 ± 0.05	1.61 ± 0.05	*p* < 0.001
Number of words per sentence	22 ± 2.1	18.6 ± 2.2	*p* < 0.0001
Flesch reading ease score	52.1 ± 5.0	45.7 ± 5.4	*p* < 0.0001

*PLI* parent information leaflet, *GFI* Gunning Fog Index, *SMOG* Simple Measure of Gobbledygook, *FKGL* Flesch Kincaid Grade Level, *CLI* Coleman–Liau Index, *ARI* Automated Readability Index.

## Discussion

It is recognised that health literacy contributes to health inequalities. Limited literacy is associated with higher healthcare costs, increased rates of hospitalisation, more access to healthcare services, and decreased use of screening and other procedures.^9^ The aim of this study was to evaluate the readability of a large cohort of paediatric PILs in order to determine whether the research portfolio of a single paediatric centre was accessible, in terms of health literacy, for the average parent. This study also investigated whether there was a difference in the readability of PIL between specialities and study subtypes.

The results from this study found that the majority of research studies fall outside of the acceptable reading age expected for a UK adult. Only one PIL achieved our predefined acceptable reading grade which is equivalent to a health literacy level of an 11–12-year old and 81% of PILs were considered to be pitched at a difficult level of reading. Further analysis demonstrated no major differences between the PILs according to whether the studies were medical or surgical in terms of their readability scores; however, the surgical PILs tended to have fewer characters and words, which may enhance their accessibility. Our findings did reveal that commercial studies were much less accessible than non-commercial studies across all of the reading tools that were used and the additional descriptors. Therefore, for clinical trials of investigational products, parents are likely to struggle to understand the information provided, and this may introduce barriers to participation and contribute to inequalities in improving health outcomes.

**Fig. 2:**
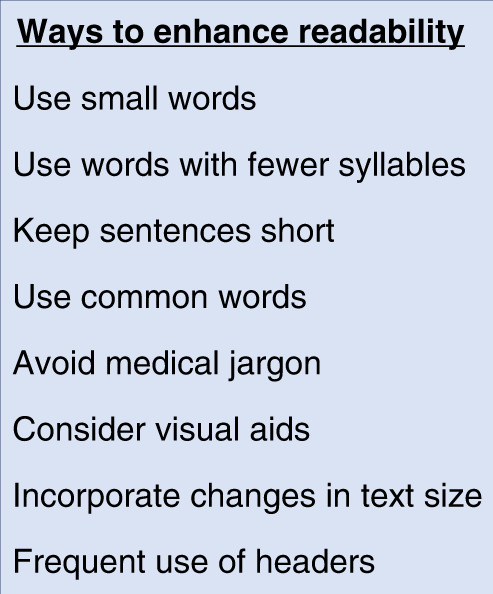
A summary of recommendations on how to improve the readibility of patient information leaflets.

In research studies, adequate informed consent is a vital principle of good clinical practice. Patients are given information which they must fully understand in order to make an autonomous decision. The most common method to provide this information is through written information leaflets.^11^ A previous study looked into the readability of 8 paediatric PILs.^12^ It reported that none of the PILs had an acceptable reading age (taken as grade 5 or less) which aligned with our findings. Another study evaluated the potential to improve the readability of materials given to patients in an ophthalmology department by calculating the FKGL scores before and after the revision of the documents.^13^ Prior to revision, the mean FKGL score was pitched at grade 11 (equivalent to 16–17 years old), and after revision, it improved to grade 6 (equivalent to 11–12 years old), suggesting that the use of readability tools is beneficial.^13^ A different study looked into the readability of patient education materials provided in a paediatric orthopaedic department.^14^ Through the analysis of 176 articles, the mean readability score was grade 10.2 and none of the articles were written at a reading grade less than 6, similar to our findings. Our study found that commercial PIL were far less accessible than non-commercial studies and this is in keeping with a previous study that looked at the difference between the readability of commercial and non-commercial cancer clinical trial websites where 6.7% of non-commercial websites were written at the recommended reading level whilst none of the commercial websites was.^15^ It also reported a higher percentage of the commercial websites were scored as difficult in terms of literacy (grade 10 or above reading level).^15^ Overall, it seems there is a consistent issue with health information being pitched above the recommended reading age. We have summarised some recommendations on how to improve the readability of patient information (Fig. 2).

Readability formulas do come with recognised limitations. They provide an estimate on readability, but they should not be taken as a measure of how well a text can be fully understood. Readability is impacted by many other factors that cannot be measured by readability formulas such as the use of visual aids, text size, use of headers and line spacing.^16^ Readability formulas estimate readability by analysing the number of syllables per word in a sentence or the average number of words per sentence but do not account for the complexity of medical vocabulary or the familiarity of the patient with medical terminology.^17^ For example, the word ‘operation’ can increase the readability grade due to its frequency of syllables; however, the general public will be more likely to understand the term when compared to a word such as ‘stent’ which has a low frequency of syllables and thus decreased readability grade.^17^ These are important considerations when aiming to improve the accessibility of written information. In addition to the limitations mentioned when using the scoring tools, this study does have its own limitations which include the single-centre site, the low number of study subtypes and the crude analysis of using only the written literature where some studies had supportive animations or websites that may be more accessible.

Our findings suggest that researchers should actively use software to estimate the readability of their material as a guide to making improvements. Possible suggestions within the literature to improve the reading accessibility of PILs include the use of simple, common words in short sentences and writing in the active voice in a conversational and personalised style.^9^ Vital information should be presented clearly and directly, and illustrations can be helpful.^17^ Audio-visuals could be used for low-literacy patients, but these would need to be carefully selected as these would have their own literacy demands.^9^ Medical professionals should be encouraged to pitch information appropriately with the support of scoring tools and perhaps presenting the materials in terms of their level of readability, for example: easy, medium, and difficult to meet individual literacy needs.^12^

## Conclusion

Accessibility to research is an important topic and it is recognised that poor literacy contributes to health inequalities. This study has demonstrated that there are significant improvements needed in the readability of PIL to allow all patients to access research studies. Regulatory boards, such as ethical approval committees, and national research organisations, such as the National Institute of Health or National Institute of Health Research, should actively encourage the use of reading age evaluation when approving or developing PIL.

## Data Availability

The materials described in the manuscript, including all relevant raw data, will be freely available to any researcher wishing to use them for non-commercial purposes, without breaching participant confidentiality. The datasets generated during and/or analysed in the current study are not publicly available due to this being a small, self-funded study but they are available from the corresponding author on reasonable request.

## References

[1] Rowlands G (2015). A mismatch between population health literacy and the complexity of health information: an observational study. Br. J. Gen. Pract..

[2] Liu L, Qian X, Chen Z, He T (2020). Health literacy and its effect on chronic disease prevention: evidence from China’s data. BMC Public Health.

[3] Berkman ND, Sheridan SL, Donahue KE, Halpern DJ, Crotty K (2011). Low health literacy and health outcomes: an updated systematic review. Ann. Intern. Med..

[4] Sharma N, Tridimas A, Fitzsimmons PR (2014). A readability assessment of online stroke information. J. Stroke Cerebrovasc. Dis..

[5] Williams, J. *The Skills for Life survey: A National Needs and Impact Survey of Literacy, Numeracy and ICT Skills* (The Stationery Office, 2003).

[6] Teravainen-Goff, A., Flynn, M., Riad, L., Cole, A. & Clark, C. Seldom-heard voices Adult literacy in the UK. *Adult Literacy report* https://cdn.literacytrust.org.uk/media/documents/Adult_Literacy_2022_report_FINAL.pdf (2022).

[7] Bostock S, Steptoe A (2012). Association between low functional health literacy and mortality in older adults: longitudinal cohort study. BMJ.

[8] Wang LW, Miller MJ, Schmitt MR, Wen FK (2013). Assessing readability formula differences with written health information materials: application, results, and recommendations. Res Soc. Adm. Pharm..

[9] McCray AT (2005). Promoting health literacy. J. Am. Med Inf. Assoc..

[10] Docimo S, Seeras K, Acho R, Pryor A, Spaniolas K (2022). Academic and community hernia center websites in the United States fail to meet healthcare literacy standards of readability. Hernia.

[11] Pandiya A (2010). Readability and comprehensibility of informed consent forms for clinical trials. Perspect. Clin. Res..

[12] Swartz EN (2010). The readability of paediatric patient information materials: are families satisfied with our handouts and brochures. Paediatr. Child Health.

[13] Williams AM, Muir KW, Rosdahl JA (2016). Readability of patient education materials in ophthalmology: a single-institution study and systematic review. BMC Ophthalmol..

[14] Delaney FT, Doinn TO, Broderick JM, Stanley E (2021). Readability of patient education materials related to radiation safety: what are the implications for patient-centred radiology care. Insights Imaging.

[15] Hillyer GC (2020). Readability of cancer clinical trials websites. Cancer Control.

[16] Plaven-Sigray, P., Matheson, G. J., Schiffler, B. C. & Thompson, W. H. The readability of scientific texts is decreasing over time. *Elife* **6**, e27725. 10.7554/eLife.27725 (2017). 10.7554/eLife.27725 PMC5584989 28873054

[17] McEnteggart GE (2015). Readability of online patient education materials related to IR. J. Vasc. Inter. Radio..

